# SARS-CoV-2 Variants BA.2.86 and EG.5.1 Alongside Scrub Typhus and Nipah in India During the Ongoing Cricket World Cup 2023: Threat Perceptions and Countermeasures

**DOI:** 10.7759/cureus.48895

**Published:** 2023-11-16

**Authors:** Ranjan K Mohapatra, Ahmed Mahal, Snehasish Mishra, Venkataramana Kandi, Wajdi J Obaidullah

**Affiliations:** 1 Department of Chemistry, Government College of Engineering, Keonjhar, Keonjhar, IND; 2 Department of Medical Biochemical Analysis, College of Health Technology, Cihan University-Erbil, Kurdistan, IRQ; 3 School of Biotechnology, Kalinga Institute of Industrial Technology (KIIT) Deemed University, Bhubaneswar, IND; 4 Department of Clinical Microbiology, Prathima Institute of Medical Sciences, Karimnagar, IND; 5 General Department of Medical Services, Ministry of Interior, Riyadh, SAU

**Keywords:** covid-19, sars-cov-2, countermeasures, threat perceptions, icc world cup 2023, infectious disease, sars-cov-2 variants

## Abstract

The United States (US), the United Kingdom (UK), and China witnessed rising cases of coronavirus disease 2019 (COVID-19) in 2023. Concerns about severe acute respiratory syndrome coronavirus 2 (SARS-CoV-2) novel strains amid the sudden surge of COVID cases are growing. Recently, BA.2.86 (Pirola) poses a much greater risk due to its higher transmission rate and spreading across regions. Pirola variant has mutations that set it apart from all earlier known SARS-CoV-2 variants. This variant was designated a variant of interest by the World Health Organization (WHO). Another SARS-CoV-2 variant named "Eris" (EG.5.1) was detected in India and started picking up in the US and the UK. The WHO listed EG.5.1 (variant) as a variant under monitoring. Therefore, it is important to remain vigilant. Further, multiple Nipah virus infections and scrub typhus cases are spreading among humans in India currently. In this situation, the 13th edition of the International Cricket Council (ICC) Men's Cricket World Cup is being held in India this year. With global reach, this big sporting carnival attracts millions of cricket fans from several countries. In light of the multiple public health concerns encountered currently, this gala global sports event needs additional preventive measures.

## Editorial

The first half of this year witnessed rising cases of coronavirus disease 2019 (COVID-19) in China, the US, and the UK and its community transmission. The main reason for this could be attributed to the emerging novel variants and subvariants of SARS-CoV-2 worldwide. Some of these novel SARS-CoV-2 variants manifest numerous mutations in the genes responsible for the spike protein. The immunity that was seemingly acquired from an earlier infection and/or vaccination, thus, could not protect from the infection. Also, the policies by the national governments to limit COVID testing would have certainly contributed to the reduced number of reported confirmed cases in the real scenario. As the existing vaccines are insufficient to provide protection against these novel variants, many countries have rolled out new vaccine doses. The BA.2.75 and XBB variants are the most noted ones worldwide. The European Centre for Disease Prevention and Control (ECDC) categorises the BA.2.75 variant under the variants of interest (VOI). BN, CH, and other sub-lineages of the BA.2.75 variant and also XBF and XBK as Omicron-Omicron recombinants sharing the same spike as BA.2.75 are monitored under BA.2.75 lineages. BA.2.86 (Pirola) poses a much greater risk as per the Centers for Disease Control and Prevention (CDC), as its transmission rate is higher across regions. Pirola variant has as many as 35 mutants setting it apart from all earlier known SARS-CoV-2 variants. Spike mutations of interest in BA.2.86 are I332V, R403K, D339H, V445H, G446S, N481K, N450D, L452W, 483del, E484K, and F486P. Without any evidence of the severity of its infection and the impact on immunity [1], the ECDC categorised this variant under the variants under monitoring (VUM). It is difficult to gauge the severity of the Pirola variant as of now due to little available samples. Pirola variant could potentially affect those who were COVID-19 infected or had received COVID-19 vaccines, as opined by some health agencies. Due to the substantially mutated spike protein of BA.2.86, concerns are being raised about its potential immunity evasion. It indicates that the existing COVID-19 vaccines may be less effective in providing protection against the variant. It also indicates high breakthrough infection potential as compared to previous SARS-CoV-2 strains.

The BA.2.86 variant was first identified in Denmark in July 2023 and was designated a VOI by the WHO. The Pirola variant cases are reportedly detected in Denmark, Canada, Portugal, Sweden, South Africa, the US, the UK, and Israel [2]. The rise in COVID-19 cases globally scares the healthcare infrastructure worldwide of another COVID-19 wave in the offing.

Another SARS-CoV-2 variant named "Eris" (EG.5.1) is also detected in India and reported. However, there has been no significant surge in infection cases since its first detection, suggesting that this subvariant has not been able to make a noticeable impact in India [3]. However, as EG.5.1 cases have started picking up in the US and the UK, it is important to remain vigilant. The WHO listed this EG.5.1 (variant) as a VUM on July 19, 2023.

A southern Indian state Kerala reported multiple Nipah virus infection cases, spreading to humans through animal (like bats and pigs) contacts. The zoonosis could spread through direct contact with infected individuals too [4]. However, the source of this current Nipah outbreak is unknown. This is the sixth Nipah outbreak seen in India. Previously, it was reported from West Bengal (2001, 66 cases, case fatality rate (CFR) = 68%), West Bengal (five cases, CFR = 100%), Kerala (2018, 23 cases, CFR = 91%), Kerala (2019, one case, survived), and Kerala (2021, one case, CFR = 100%) [5]. In the prevailing situation, scrub typhus (another infectious disease) cases are also rising in Indian states like Rajasthan, Odisha, Himachal Pradesh, Maharashtra, and Telangana. The scrub typhus cases are also reportedly increasing alongside dengue and flu in some states. The combined flu, scrub typhus, or typhoid cases alongside dengue obscures healthcare initiatives, especially among children, the immunocompromised, and the elderly.

In this situation, the 13th edition of the International Cricket Council (ICC) Men's Cricket World Cup is being held in India this year, slated to be played between 5th October and 19th November. With global reach, Australia, New Zealand, South Africa, England, the Netherlands, Pakistan, Afghanistan, Bangladesh, and Sri Lanka are the nine countries participating along with India (the host, as the 10th country) in this big sporting carnival. Matches are being played in several densely populated metro cities across the country. Cricket is a very popular sport globally, including in India, and its popularity will expectedly rise further with its recent inclusion in the Olympics. As a result, millions of cricket fans from different participating and non-participating countries throng the stadia [6]. In light of the multiple public health concerns encountered currently as discussed above, this gala global sports event needs additional preventive measures. Mass gatherings in popular sports events like cricket could be risky in the current situation. The "new normal" scenario in the ongoing pandemic despises that mass gatherings could potentially pose serious health issues facilitating the transmission and spread of infectious diseases. Also, winter is arriving in the region soon. So, the risks of transmissible diseases like winter flu, hepatitis A and B, diarrhoea, and measles among the thick-floating population cannot be ruled out [6,7], especially amid the resurging COVID-19 cases. Foolproof cautionary measures during the ongoing 2023 ICC Men's Cricket World Cup may be ensured among the fans, spectators, and sports enthusiasts, being a potential superspreader. Further, the globalisation of such infectious diseases among the players and the spectators cannot be ruled out when this mega event is over.

The risk is further increased with comorbidities like age, cardiac health, diabetes, chronic kidney disease, and pulmonary history of asthma and tuberculosis. As per health reports, India has a high population with these risks. This high-risk population may need to remain alert and take precautions like regular hand hygiene, respiratory hygiene, ventilated house and workplace, and face-masking, particularly in crowded areas, chiefly owing to the fact that India reportedly had poor booster dose coverage during the COVID-19. Abiding by COVID-appropriate behaviour near the venue/stadia and elsewhere is recommended as the available vaccines seem to be low on efficiency against the emerging subvariants [8].

Conclusion

Although regular high-level meetings are needed to review the global and national COVID-19 situation, novel circulating variants and their public health impact, with denuding contact-tracing and testing day-by-day and almost zero surveillance in place, predicting how and when the new SARS-CoV-2 variants could spread may be difficult. England has planned to start the flu and COVID-19 vaccine programme earlier as a precautionary measure after the new SARS-CoV-2 variant was recently identified. Even though therapeutics seem to address severe clinical conditions, hospitalisation, and critical situations well, booster vaccination against the variants and subvariants of SARS-CoV-2 is suggested as an additional shield. Nevertheless, developing more efficient vaccines and monoclonal antibodies (mAbs) is ever more urgent. It is crucial to strictly implement non-discriminatory traveller guidelines. Genomic surveillance targeted at international travellers could help. Practically feasible COVID-appropriate social distancing, hand-sanitising and face-masking, and "test and monitor" measures are recommended, while surveillance and monitoring of urban water management systems shall facilitate curbing health emergencies as an early warning tool.
